# HBsAg glycan isomer: a potential circulating biomarker for monitoring cccDNA and predicting virologic response in chronic hepatitis B

**DOI:** 10.3389/fcimb.2026.1818958

**Published:** 2026-08-20

**Authors:** Jin Li, Zhiqing Hu, Min Wu, Yuanlan Ji, Xiaonan Zhang, Fan Chen, Xunxun Wu, Qiongyue Zhang, Cailin Chen, Chuanwu Zhu, Li Zhu

**Affiliations:** 1 Department of Infectious Diseases, The Affiliated Infectious Diseases Hospital of Soochow University, Suzhou, Jiangsu, China; 2 Shanghai Public Health Clinical Center, Fudan University, Shanghai, China; 3 School of Life Sciences, Technical University of Munich, Freising, Germany; 4 Department of Gastroenterology, The Affiliated Jiangsu Shengze Hospital of Nanjing Medical University, Suzhou, Jiangsu, China

**Keywords:** biomarker, cccDNA, CHB, HBsAgGi, transcriptional activity

## Abstract

**Background:**

Previous research has identified that Hepatitis B surface antigen glycan isomer (HBsAgGi) specifically marks O-glycosylated M-HBsAg exclusively present on infectious Dane particles (unlike total HBsAg, which is dominated by non-infectious subviral particles), it offers a more specific association with infectious viral burden and cccDNA activity. Accordingly, the present study aims to assess the potential of HBsAgGi as a circulating biomarker to reflect intrahepatic covalently closed circular DNA (cccDNA) and transcriptional activity, and for monitoring antiviral response.

**Methods:**

A total of 73 individuals diagnosed with chronic genotype C HBV infection were recruited for this study. Serum concentrations of HBsAgGi, HBsAg, HBeAg, HBV DNA, and HBV RNA were measured. Intrahepatic cccDNA and HBV RNA loads were determined in liver biopsy specimens obtained from 16 patients. Correlations between serum HBsAgGi and intrahepatic markers were analyzed. We measured serum Hepatitis B core-related antigen (HBcrAg) levels in 28 patients. 18 patients received nucleos(t)ide analogue therapy for 48 weeks and we evaluated levels of HBsAgGi after therapy. Additionally, receiver operating characteristic (ROC) curve analysis was conducted to assess the discriminative ability of the candidate biomarkers in predicting virological response (VR).

**Results:**

Serum HBsAgGi demonstrated significant positive correlations with intrahepatic cccDNA (r = 0.7172, *P* < 0.01), HBV RNA (r = 0.7879, *P* < 0.01), and the RNA/cccDNA ratio (r = 0.5840, *P* < 0.05), showing stronger correlations than conventional serum markers (HBcrAg, HBV RNA, DNA, HBsAg). HBsAgGi also showed the strongest correlation with serum HBcrAg (r = 0.5361, *P* < 0.01) in 28 patients. During treatment, HBsAgGi levels declined and baseline HBsAgGi predicted VR with superior accuracy (AUC = 0.9538) versus other markers.

**Conclusion:**

Serum HBsAgGi represents an emerging, minimally invasive serum indicator for assessing intrahepatic cccDNA loads and viral transcriptional activity, demonstrating stronger correlations with intrahepatic markers compared to conventional serum markers in this cohort. It also shows promising utility for predicting treatment response.

## Introduction

Chronic hepatitis B virus (HBV) infection, affecting approximately 240 million individuals worldwide, remains a major global public health burden. As a primary driver of liver cirrhosis and hepatocellular carcinoma (HCC), this infection accounted for an estimated 1.1 million deaths in 2024 (WHO, 2026). Current antiviral strategies for chronic hepatitis B (CHB) is predominantly centered on prolonged nucleos(t)ide analogue (NA) administration or time-limited 48-week courses of pegylated interferonalfa2a (Peg-IFNα) (Terrault et al., 2016; Terrault et al., 2018; Ghany et al., 2026). Nevertheless, treatment responses exhibit marked heterogeneity among patients, while the barrier to a full cure is largely attributed to the resilience of covalently closed circular DNA (cccDNA), the intranuclear transcriptional template refractory to existing therapies (Nassal, 2015; Charre et al., 2019; van Bömmel and Berg, 2021). While current clinical practice utilizes routine markers like HBsAg and HBV DNA for monitoring of disease, intrahepatic cccDNA abundance and transcriptional activity represent the gold standard for assessing therapeutic efficacy and predicting sustained off-treatment responses - attributable to its role as the foundational HBV replication template (Charre et al., 2019; van Bömmel and Berg, 2021). However, the invasiveness of biopsy-dependent cccDNA quantification precludes routine clinical implementation (Tu et al., 2022). This critical limitation underscores an urgent need for serum biomarkers that accurately mirror transcriptionally active cccDNA reservoirs, surpassing conventional tools. Recently, novel serological markers, including HBV RNA and hepatitis B core-related antigen (HBcrAg), have emerged as surrogate indicators for monitoring cccDNA kinetics, aiming to enable precision monitoring, guide personalized therapy, and advance curative strategies (Chen et al., 2019; Testoni et al., 2024; Wang et al., 2024).

Recently, the HBsAgGi assay, a quantitative method for assessing O-glycosylation levels within the PreS2 domain of M-HBsAg in genotype C HBV, has recently been established by investigators in Japan (Wagatsuma et al., 2018; Angata et al., 2022). Since O-glycosylated M-HBsAg is exclusively present on infectious HBV particles, known as Dane particles, and absent from non-infectious subviral particles (SVPs), which are found in the serum of CHB patients at approximately 1000 times the number of Dane particles (Bruss, 2007; Schädler and Hildt, 2009), HBsAgGi has a more specific association with infectious particles compared to the routine clinical marker, total serum HBsAg. Consequently, HBsAgGi is more suitable for accurately assessing the infectious viral burden. Initial investigations regarding HBsAgGi have revealed notable associations with conventional HBV indicators, such as HBsAg, HBV DNA, and HBeAg, alongside emerging marker like HBV RNA and HBcrAg (Murata et al., 2022; Okumura et al., 2023; Ikeda et al., 2024; Yao et al., 2025). Additionally, HBsAgGi has shown potential as an independent prognostic factor for HBsAg seroclearance and hepatocellular carcinoma (HCC) onset in patients undergoing NA treatment (Okumura et al., 2023; Ikeda et al., 2024; Kozuka et al., 2024). Notably, unlike total serum HBsAg, which can be produced from both cccDNA and integrated HBV DNA, HBsAgGi production is indicated hypothesized to be driven primarily by transcriptionally active cccDNA rather than integrated HBV DNA. This unique characteristic positions HBsAgGi as a potentially more accurate non-invasive surrogate for reflecting intrahepatic cccDNA reservoirs and their transcriptional activity.

The association linking serum HBsAgGi levels to intrahepatic cccDNA loads and transcriptional status remains unexplored. In the present study, serum HBsAgGi concentrations were quantified and evaluated in a cohort of genotype C CHB individuals recruited from Suzhou, China. We focused on examining the relationship between serum HBsAgGi and intrahepatic cccDNA levels, as well as the HBV RNA/cccDNA ratio, a direct marker of cccDNA transcriptional activity. Additionally, we compared these findings with conventional serum HBV markers and emerging biomarkers such as HBV RNA and HBcrAg. Furthermore, using longitudinal data from baseline and week 48 of nucleos(t)ide analogue (NA) therapy, we analyzed the effectiveness of baseline HBsAgGi in predicting antiviral treatment response and compared it with other predictors.

## Materials and methods

### Study design and patient cohorts

The cross-sectional observational study with prospective sample collection and retrospective analysis on stored samples was conducted on 73 individuals diagnosed with genotype C CHB who underwent routine monitoring at the Fifth People’s Hospital of Suzhou from March 2021 to June 2024. Inclusion criteria required confirmed HBsAg positivity persisting for over 6 months. Exclusion criteria encompassed: (1) current or previous engagement in antiviral therapy; (2) concurrent chronic liver conditions, such as hepatitis C, alcoholic liver disease, non-alcoholic fatty liver disease, primary biliary cholangitis, autoimmune hepatitis, or drug-induced liver injury; (3) serious systemic comorbidities, including HIV infection or malignancies; (4) pregnancy or lactation; (5) administration of immunosuppressive therapy within the preceding 6 months. Serum specimens were obtained during the initial clinical visit and preserved at -80 °C pending analysis. Ethical approval was granted by the Ethics Committee of the Fifth People’s Hospital of Suzhou, adhering to the principles of the Helsinki Declaration (Approval No. SZWY2023007). Patient confidentiality was maintained, and written informed consent was secured from all participants.

A total of 73 untreated patients with genotype C CHB infection constituted the overall cohort. Based on the availability of clinical data and biological samples, this cohort was stratified into three specific subcohorts for distinct analyses (**Supplementary Figure 1**): (1) The HBcrAg and Liver Biopsy Subcohorts: A total of 28 patients with available baseline serum samples were included in the HBcrAg subcohort for serum viral marker quantification. Within this group, a subset of 16 patients who underwent liver biopsy constituted the Liver Biopsy sub-cohort. These 16 patients provided paired liver tissue specimens and contemporaneous serum samples obtained at the time of biopsy, which were used for the analysis of intrahepatic HBV markers. (2) The Treatment Subcohort: This group included 18 patients who subsequently initiated NA therapy and had complete paired serum samples available at both baseline and week 48 of therapy. The treatment regimens included Entecavir (ETV, n=3), Tenofovir alafenamide (TAF, n=9), Tenofovir disoproxil fumarate (TDF, n=1), and Tenofovir amibufenamide (TMF, n=5). All patients in this subcohort received treatment for a minimum of 48 weeks. Virological response (VR) was defined as serum HBV DNA levels falling below the lower limit of detection (<20 IU/mL) at week 48 of treatment. (3) Remaining Patients: The remaining 27 patients from the overall cohort were not included in the specific sub-analyses mentioned above. These patients were excluded from specific sub-cohorts because they lacked paired liver tissue specimens, did not undergo serum HBcrAg quantification, and/or did not receive subsequent NA therapy with available archived serum samples.

### Serum biochemical and virological markers

Serum biochemical profiles, including alanine aminotransferase (ALT) and aspartate aminotransferase (AST), were assessed via an automated chemistry analyzer (Hitachi 7600-110; Hitachi, Tokyo, Japan). Complete blood counts, including platelet (Plt) counts, were measured using an XT-4000i automated hematology analyzer (Sysmex Corporation, Kobe, Japan). Quantification of HBV DNA loads was performed using real-time PCR (COBAS TaqMan HBV Test v2.0; Roche Diagnostics, Mannheim, Germany). Serum HBsAg concentrations and HBeAg status were evaluated with the Abbott Architect immunoassay system (Abbott Laboratories, Abbott Park, IL, USA). HBV RNA isolation from 250 μL serum was carried out using the HBV RNA Assay Kit (Rendu Biotech, Shanghai, China) in strict accordance with the manufacturer’s guidelines. HBcrAg quantification was conducted via ultrasensitive chemiluminescent enzyme immunoassay (CLEIA) (Fujirebio Inc., Tokyo, Japan).

### HBV genotyping

HBV genotyping (genotypes A through F) was determined through a nested PCR assay utilizing type-specific primers targeting the pre-S/S gene region, as previously described (Naito et al., 2001). The first-round PCR used universal outer primers (P1 and S1-2) with standard thermocycling conditions (40 cycles, 55 °C annealing). The second-round PCR used the product of the first-round PCR and two primer mixtures: Mix A (common primer B2 and type-specific primers for genotypes A, B, C) and Mix B (common primer B2R and type-specific primers for genotypes D, E, F). The second-round amplification involved a two-step annealing protocol (20 cycles at 58 °C followed by 20 cycles at 60 °C). The specific genotypes were identified based on the distinct sizes of the genotype-specific DNA bands visualized by 3% agarose gel electrophoresis. The detailed primer sequences and specific thermocycling conditions are fully described (Naito et al., 2001).

### Intrahepatic cccDNA and HBV RNA quantification

For the quantification of intrahepatic cccDNA and HBV RNA, total nucleic acids were isolated from liver biopsy specimens via phenol-chloroform extraction. Prior to cccDNA amplification, total DNA underwent digestion with Exonuclease I (Exo I) and Exonuclease III (Exo III) to selectively eliminate non-cccDNA HBV structures, such as relaxed circular DNA (rcDNA) and duplex linear DNA (dlDNA). Then cccDNA was amplified by real-time qPCR utilizing the following primers and probe: cccDNA forward primer 5’-CGTCTGTGCCTTCTCRTCTG-3’ (R: A/G), cccDNA reverse primer 5’-GCACAGCTTGGAGGCTTGAA-3’, and cccDNA probe 5’-FAM-ACCAATTTATGCCTACAGCCTCC-MGB-3’. To determine the cccDNA level per cell, we determined genomic DNA (gDNA) copy numbers by qPCR using the single-copy gene ‘14q24.1’ as an endogenous reference. Cellular density was calculated by dividing gDNA copies/µL by 2 (accounting for diploid genome). Finally, cccDNA level per cell were derived by dividing cccDNA copies/µL by the cellular count/µL. Total RNA was treated by dsDNase to eliminate genomic DNA and then quantified by reverse transcription-PCR (RT-PCR) using the Maxima H Minus First Strand cDNA Synthesis Kit with dsDNase (Thermo Fisher Scientific, Waltham, MA, USA). GAPDH RNA quantification functioned as an internal control for intrahepatic HBV RNA copies/cell. The limit of detection (LOD) for the intrahepatic HBV RNA assay was established at 1×10^-3^ copies/cell. cccDNA transcriptional activity was calculated as the ratio of intrahepatic RNA to cccDNA. For presentation purposes, this ratio was multiplied by ten and subjected to log10 transformation.

### Serum HBsAgGi quantification

Quantification of serum HBsAgGi was accomplished using a commercial HBsAgGi ELISA kit (RCMG Inc., Japan) adhering to the manufacturer’s instructions. In brief, aliquots of 100 μL containing 100-fold diluted patient serum and serially diluted standard protein (recombinant M-HBsAg) were introduced into each well of a 96-well plate pre-coated with monoclonal antibodies targeting O-glycosylated PreS2 on M-HBsAg. The principle of this specific antibody capture, which distinguishes infectious Dane particles from abundant SVPs, is illustrated in **Supplementary Figure 2**. Following a 2-hour incubation at ambient temperature, the plate underwent washing. Subsequently, 100 μL of HRP-conjugated HBsAgGi detection antibody was dispensed into each well and incubated for 1 hour at room temperature. After further washing steps, 100 μL of HRP substrate (TMB solution) was added to each well. The enzymatic reaction was halted after the specified incubation period by the addition of stop solution. Optical density was read at 450 nm utilizing an ELISA microplate reader. Serum HBsAgGi concentrations were derived from the standard curve constructed using diluted M-HBsAg calibrators and adjusted for sample dilution factors. According to the manufacturer’s specifications, the lower limit of detection (LLD) for M-HBsAg is 0.89 ng/mL, and the intra-assay reproducibility is demonstrated with coefficient of variation (CoV) values of less than 10% across different concentration levels.

### Statistical analyses

Data analysis was conducted utilizing GraphPad Prism 9.0, SPSS 27.0, and the Wei Sheng Xing online platform. Prior to statistical evaluation, data for serum HBsAgGi, HBsAg, HBeAg, HBV DNA, HBV RNA, HBcrAg, and intrahepatic cccDNA and HBV RNA underwent log10 transformation. Specimens exhibiting serum HBV DNA concentrations below 20 IU/mL were assigned a value of “0” to enable statistical computation. Categorical data are presented as proportions and were compared across groups via the Chi-square test. Continuous variables are described as median (interquartile range, IQR) and were examined using the Mann-Whitney U test for comparisons between groups. Intragroup variations in continuous data pre- and post-treatment were evaluated utilizing the Wilcoxon signed-rank test. Correlations between serum HBsAgGi and intrahepatic cccDNA loads or serum HBV markers (HBsAg, HBV DNA, HBV RNA, HBcrAg) were assessed through Spearman’s rank correlation analysis. The predictive efficacy of serum HBsAgGi, HBsAg, HBV DNA, and HBV RNA for antiviral treatment response was examined using Receiver operating characteristic (ROC) curves. A two-tailed P-value < 0.05 was considered statistically significant.

## Results

### Baseline characteristics and correlations of serum HBsAgGi with virological markers

**Figure 1:**
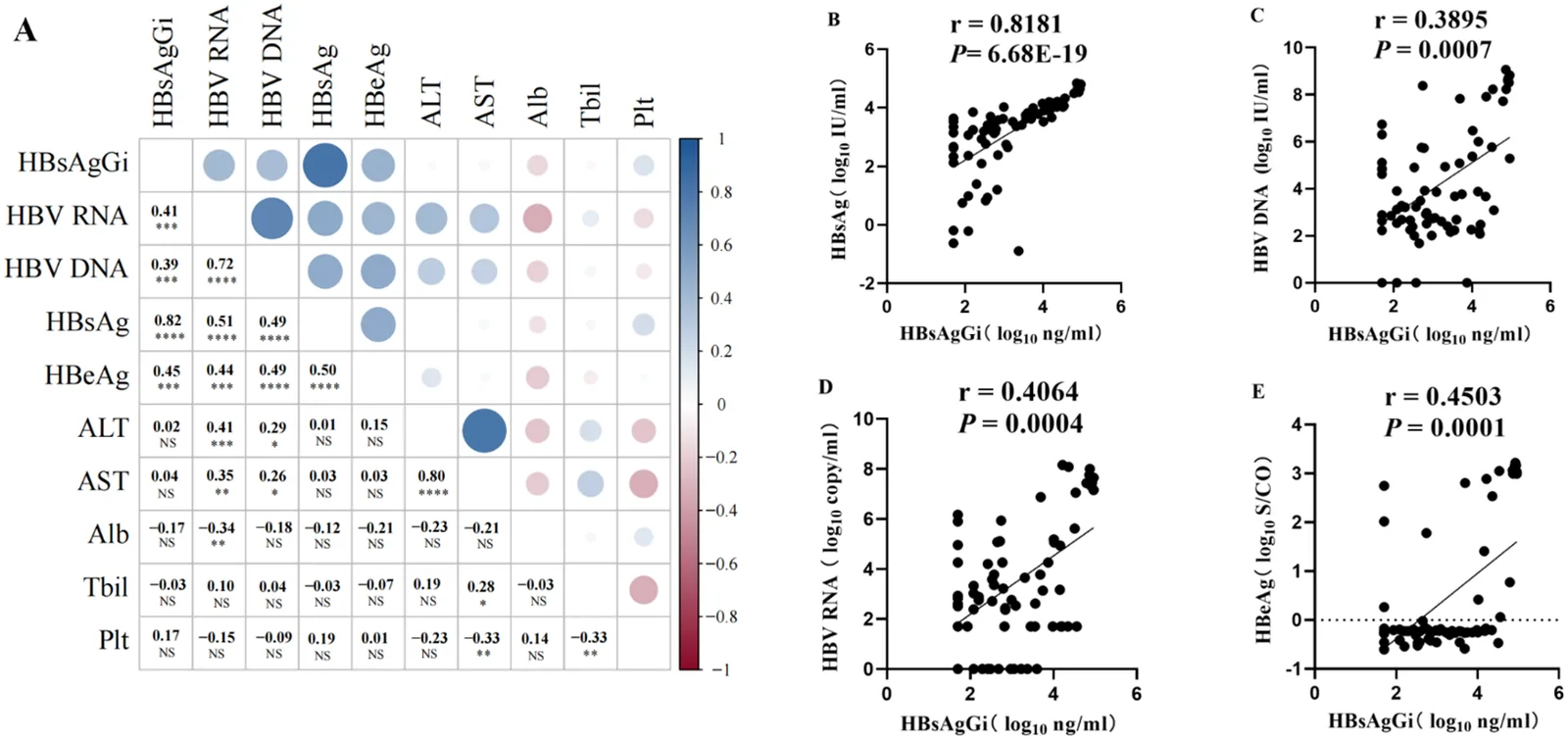
Correlation analysis of serum HBsAgGi with other virological markers and clinical parameters (n=73). **(A)** Correlation matrix heatmap illustrating the associations among serum HBsAgGi, HBV RNA, HBV DNA, HBsAg, HBeAg, and liver function indicators (ALT, AST, Alb, Tbil, Plt). The color scale represents the correlation coefficient (r), with blue indicating positive correlations and red indicating negative correlations. The lower triangle displays the specific r values and statistical significance levels (*P < 0.05, **P < 0.01, ***P < 0.001, ****P < 0.0001; NS, not significant, *P* ≥ 0.05). **(B–E)** Scatter plots detailing the correlations between serum HBsAgGi and conventional HBsAg **(B)**, HBV DNA **(C)**, HBV RNA **(D)**, and HBeAg **(E)**. The correlation coefficients r value and corresponding P-value are displayed in each panel.

The demographic and baseline clinical characteristics of the 73 enrolled patients with genotype C chronic hepatitis B are summarized in **Table 1**. Overall, 27.4% (20/73) of the cohort was HBeAg-positive. Stratified analysis revealed that serum HBsAgGi levels were significantly higher in HBeAg-positive individuals compared to their HBeAg-negative counterparts (**Table 1**). To evaluate the relationships between serum HBsAgGi and standard virological and biochemical markers, a pairwise correlation matrix was generated. Overall, HBsAgGi levels demonstrated robust positive associations with key virological markers (**Figure 1A**). The strongest correlation was observed with HBsAg, followed by moderate yet significant correlations with HBV DNA, HBV RNA, and HBeAg (**Figures 1B–E**). In contrast, no statistically significant associations were observed between HBsAgGi and liver biochemical parameters such as ALT or AST (**Figure 1A**). Subgroup analysis stratified by HBeAg status further elucidated these patterns: in HBeAg-positive individuals, HBsAgGi correlated significantly with all four virological markers (**Supplementary Figures 3A–D**). Conversely, among HBeAg-negative subjects, HBsAgGi exhibited a significant positive correlation solely with HBsAg (*P* < 0.0001; **Supplementary Figure 4A**), but not with HBV DNA, HBV RNA, or HBeAg (*P* ≥ 0.05; **Supplementary Figures 4B–D**).

**Table 1:** Baseline characteristics of 73 patients.

Characteristic	All patients (n=73)	HBeAg positive (n=20)	HBeAg negative (n=53)	*P*-value
Age (years)	41 (35 - 50)	40 (36 - 43)	42 (35 - 51)	0.2203^a^
Sex (male)	46 (63.0%)	10 (50%)	36 (67.9%)	0.1571^b^
ALT (U/L)	31 (19.5 - 56)	33.5 (28 – 75.75)	27 (19 - 54)	0.0971^a^
AST (U/L)	24 (18.5 - 34)	25 (19 – 48.25)	23 (18 - 31)	0.3498^a^
Albumin (g/L)	45.6 (43.4 - 46.35)	44.05 (39.9 - 45.6)	45.8 (44.4 - 47.25)	**0.0066** ^a^
Total bilirubin (µmol/L)	14.6 (11.4 - 19.25)	14.55 (11.08 - 17.58)	15.3 (11.55 - 19.55)	0.6561^a^
Platelet count (×10^9^/L)	200 (157 - 253)	204.5 (141 - 248)	198 (160.5 - 253.5)	0.9099^a^
HBsAg (log_10_ IU/ml)	3.53 (2.72 - 4.03)	4.28 (3.65 - 4.65)	3.24 (2.36 - 3.77)	**<0.0001** ^a^
HBV DNA (log_10_ IU/ml)	3.49 (2.53 - 5.74)	7.87 (6.07 - 8.52)	2.89 (2.26 - 3.89)	**<0.0001** ^a^
HBV RNA (log_10_ copies/ml)	3.14 (2.00 - 5.15)	7.23 (5.91 - 7.64)	2.62 (2.00 - 3.47)	**<0.0001** ^a^
HBsAgGi (log_10_ ng/ml)	2.99 (2.24 - 4.16)	4.55 (3.77 - 4.92)	2.77 (2.20 - 3.56)	**<0.0001** ^a^
HBeAg (log_10_ S/CO)	-0.22 (-0.27 - 0.38)	2.85 (1.50 - 3.05)	-0.24 (-0.40 - -0.21)	**<0.0001** ^a^

Data are expressed as median (interquartile range, IQR), except for sex which is expressed as number (percentage). HBsAgGi, hepatitis B surface antigen glycan isomer; ALT, alanine aminotransferase; AST, aspartate aminotransferase; HBsAg, hepatitis B surface antigen; HBeAg, hepatitis B e antigen.

^a^ Mann Whitney U test.

^b^ Chi-square test. Bold values indicate statistical significance (p < 0.05).

### Serum HBsAgGi as a potential non-invasive surrogate for intrahepatic HBV reservoir and transcriptional activity

**Figure 2:**
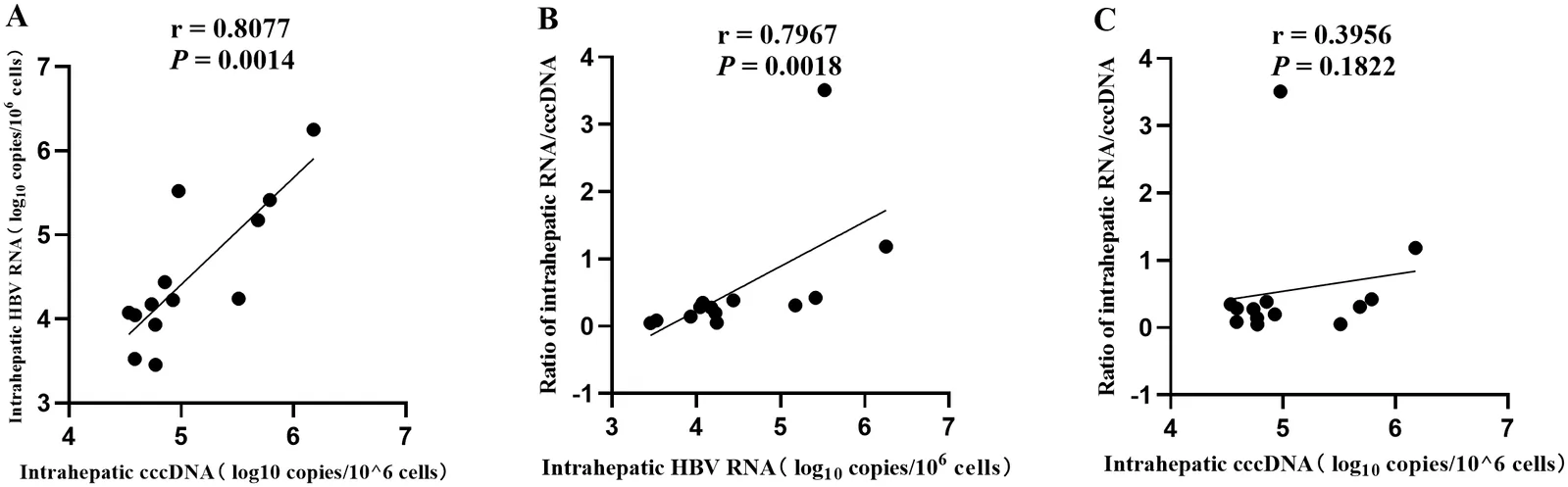
Correlations among intrahepatic HBV markers. Scatter plots showing Spearman correlation analyses of intrahepatic viral markers. **(A)** Correlation between intrahepatic HBV RNA and cccDNA levels. **(B)** Association between the ratio of intrahepatic RNA/cccDNA (a surrogate marker for viral transcriptional activity) and intrahepatic HBV RNA levels. **(C)** Association between the intrahepatic RNA/cccDNA ratio and intrahepatic cccDNA levels. All measurements are expressed as log_10_ copies/10^6^ cells. Correlation coefficients (r) and P-values are shown in each panel.

The epidemiological and clinical characteristics of the 16 patients who underwent baseline liver biopsy are summarized in **Supplementary Table 1**. The detailed median levels of serum HBsAgGi, intrahepatic cccDNA, and intrahepatic HBV RNA for this subcohort are also presented (**Supplementary Table 1**). We firstly characterized the dynamics of intrahepatic HBV markers. Intrahepatic HBV RNA levels exhibited a strong positive correlation with cccDNA levels (**Figure 2A**). Furthermore, viral transcriptional activity was significantly associated with intrahepatic HBV RNA but showed no significant correlation with cccDNA levels (**Figures 2B, C**). Collectively, these findings suggest that intrahepatic HBV RNA levels are closely linked to both cccDNA abundance and transcriptional activity.

**Figure 3:**
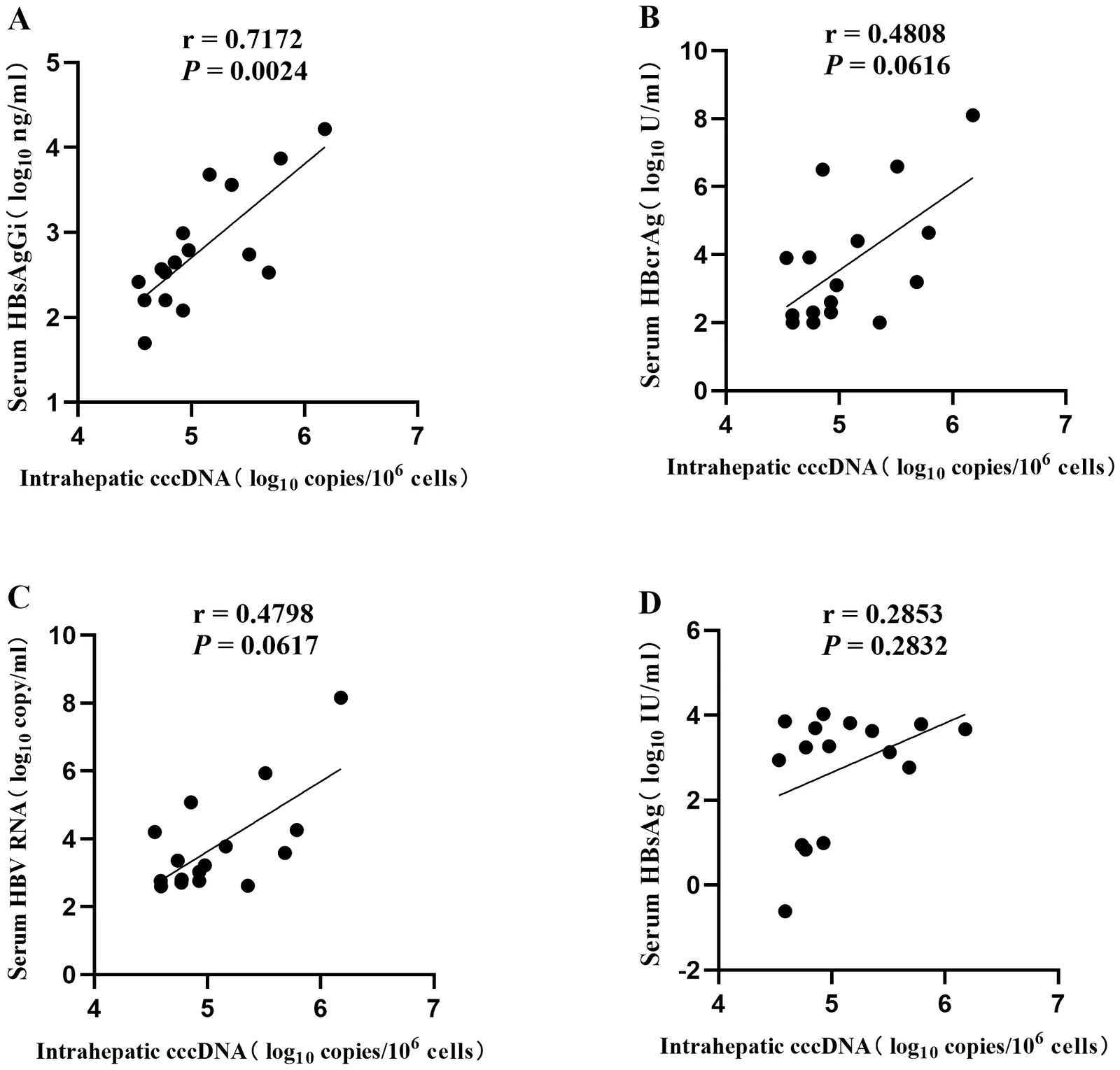
Correlations between serum viral markers and intrahepatic cccDNA levels. Scatter plots illustrating the associations of intrahepatic cccDNA levels with serum HBsAgGi **(A)**, HBcrAg **(B)**, HBV RNA **(C)**, and conventional HBsAg **(D)**. All measurements are expressed as log_10_ values. Correlation coefficients (r) and P-values are displayed in each panel.

**Figure 4:**
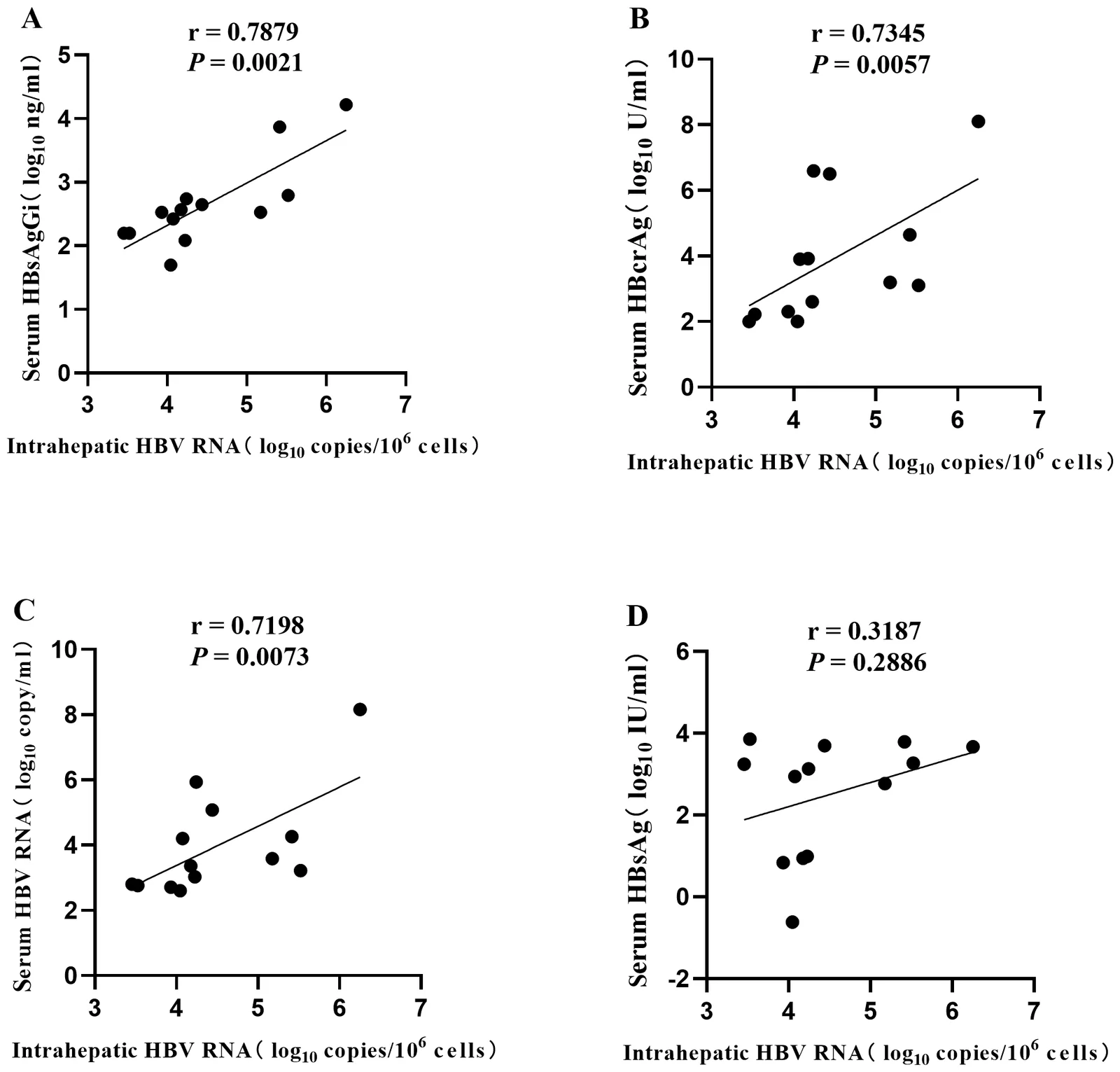
Correlations between serum viral markers and intrahepatic HBV RNA levels. Scatter plots illustrating the associations of intrahepatic HBV RNA levels with serum HBsAgGi **(A)**, HBcrAg **(B)**, HBV RNA **(C)**, and conventional HBsAg **(D)**. All measurements are expressed as log_10_ values. Correlation coefficients (r) and P-values calculated by Spearman correlation analysis are displayed in each panel.

**Figure 5:**
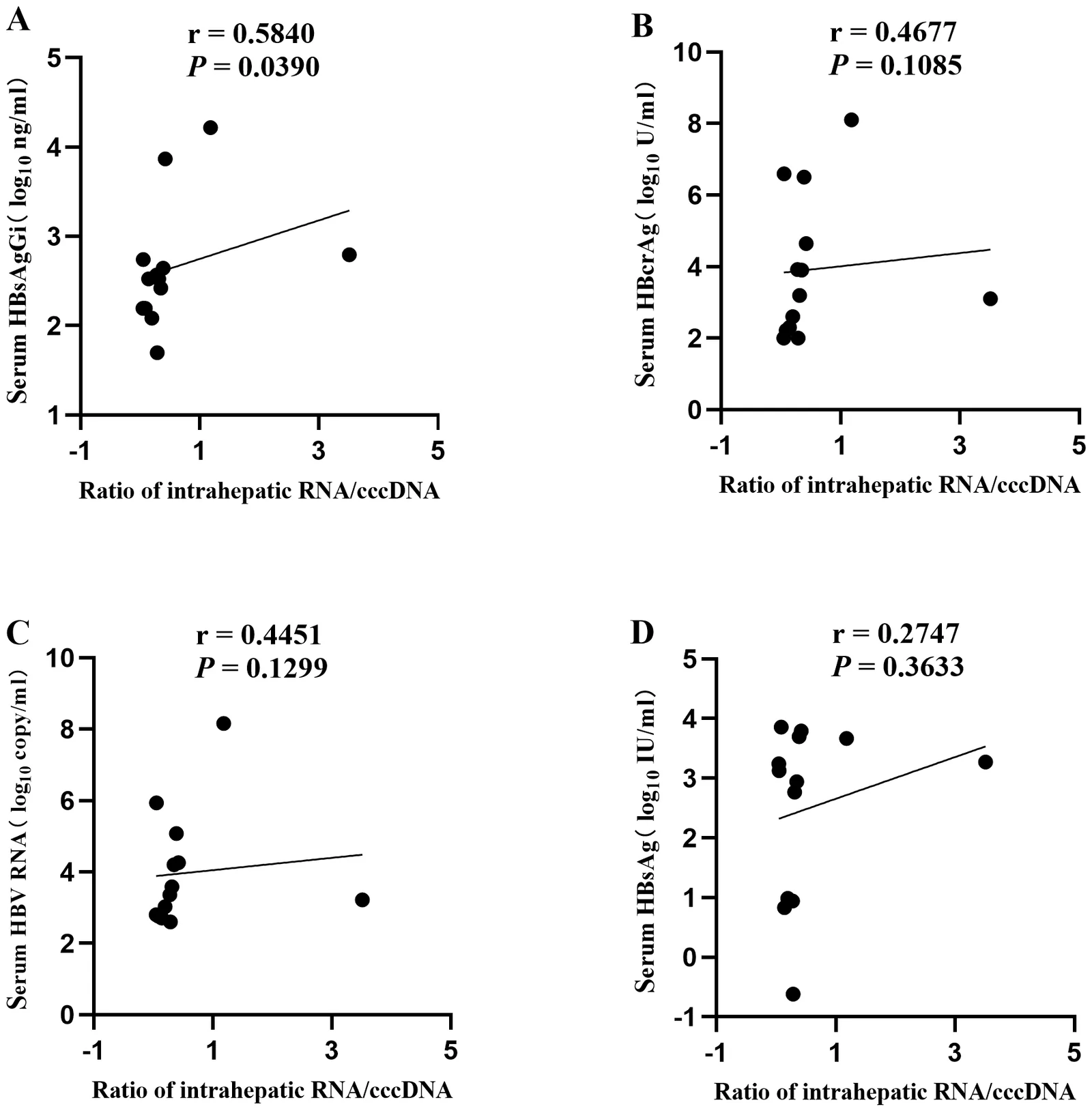
Correlations between serum viral markers and intrahepatic viral transcriptional activity. Scatter plots illustrating the associations of the intrahepatic RNA/cccDNA ratio (a surrogate marker for viral transcriptional activity) with serum HBsAgGi **(A)**, HBcrAg **(B)**, HBV RNA **(C)**, and conventional HBsAg **(D)**. All measurements are expressed as log_10_ values. Correlation coefficients **(r)** and P-values calculated by Spearman correlation analysis are displayed in each panel.

Next, we evaluated the reliability of various serum markers in reflecting the intrahepatic viral reservoir. Serum HBsAgGi demonstrated the strongest and most consistent positive correlations with both intrahepatic cccDNA and intrahepatic HBV RNA (**Figures 3A**, **4A**). While serum HBcrAg and serum HBV RNA also showed significant associations with intrahepatic HBV RNA (**Figures 4B, C**), their correlations with intrahepatic cccDNA were notably weaker or marginal (**Figures 3B, C**). Conventional serum HBsAg and HBV DNA failed to show any significant association with either intrahepatic cccDNA or HBV RNA (**Figures 3D**, **4D**; **Supplementary Figure 5A, B**). Finally, to determine which serum marker best reflects viral transcriptional activity, we analyzed correlations with the intrahepatic RNA/cccDNA ratio. Notably, serum HBsAgGi was the only marker that showed a significant positive correlation with this ratio (**Figure 5A**). In contrast, serum HBcrAg, serum HBV RNA, and conventional serum HBsAg, HBV DNA showed no significant associations (**Figures 5B–D**; **Supplementary Figure 5C**). Collectively, these preliminary findings suggest the potential of serum HBsAgGi as a promising non-invasive candidate for reflecting the intrahepatic HBV reservoir and its transcriptional activity, warranting further validation in larger cohorts.

### Serum HBsAgGi exhibits the strongest correlation with HBcrAg among serum markers

**Figure 6:**
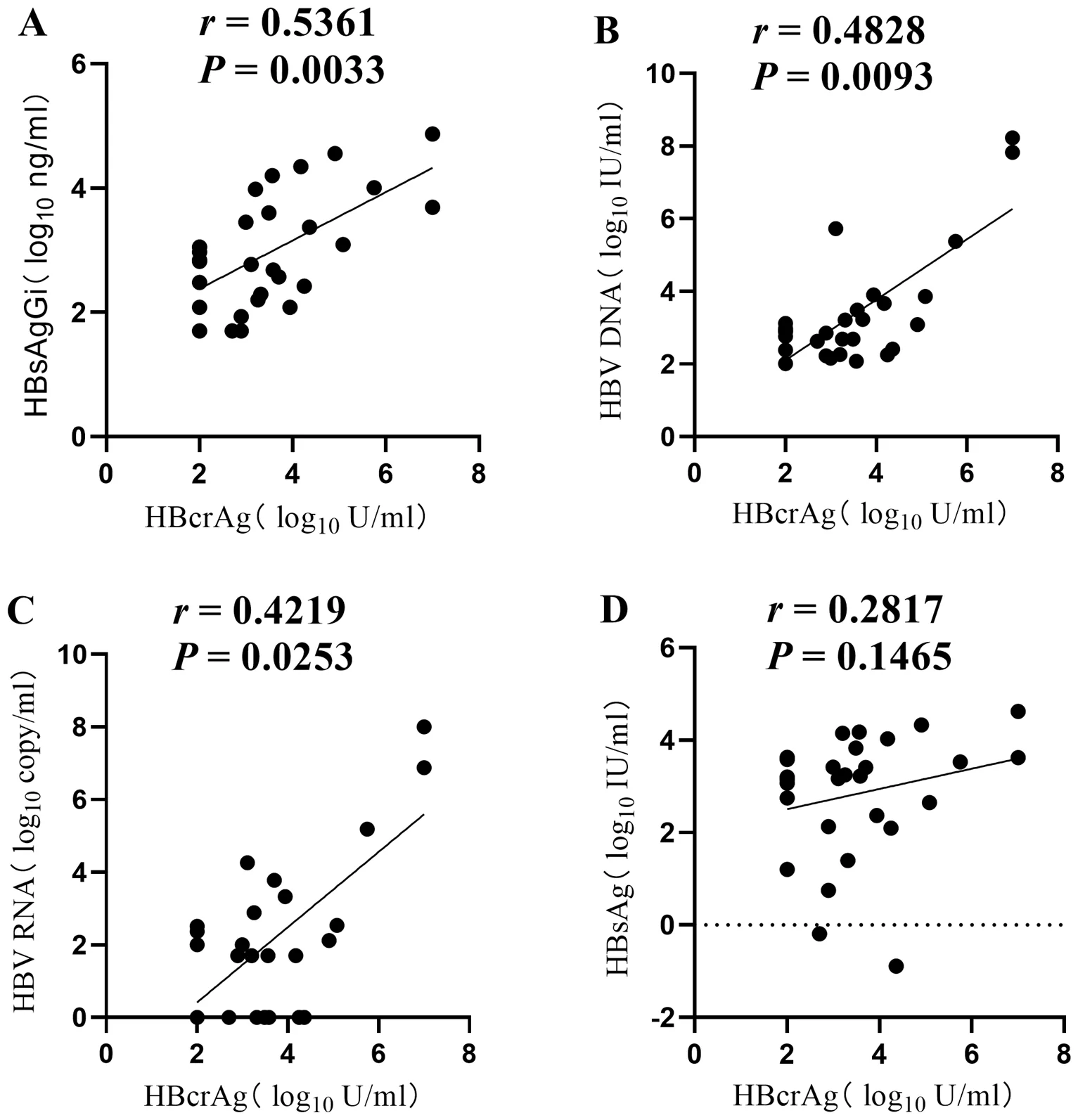
Correlations between serum HBcrAg and other serum viral markers. Scatter plots illustrating the associations of serum HBcrAg with HBsAgGi **(A)**, HBV DNA **(B)**, HBV RNA **(C)**, and conventional HBsAg **(D)**. All measurements are expressed as log_10_ values. Correlation coefficients (r) and P-values calculated by Spearman correlation analysis are displayed in each panel.

Considering serum HBcrAg as a validated proxy for intrahepatic cccDNA dynamics, we further examined its correlation with serum HBsAgGi in this study. Of the HBcrAg sub-cohort, 28 patients with available baseline HBcrAg data were included for this analysis, and their demographic and clinical characteristics are summarized in **Supplementary Table 2**. Spearman correlation analysis identified a robust positive association between serum HBsAgGi and HBcrAg (**Figure 6A**). Importantly, this correlation was notably stronger than the associations between HBcrAg and other virological markers, including HBV DNA and HBV RNA (**Figures 6B, C**). Furthermore, no significant association was observed between HBcrAg and conventional HBsAg (**Figure 6D**). These findings suggest that HBsAgGi shares the closest relationship with HBcrAg among the evaluated serum markers.

### Longitudinal changes of serum HBsAgGi and its correlations with virological markers during NA therapy

**Figure 7:**
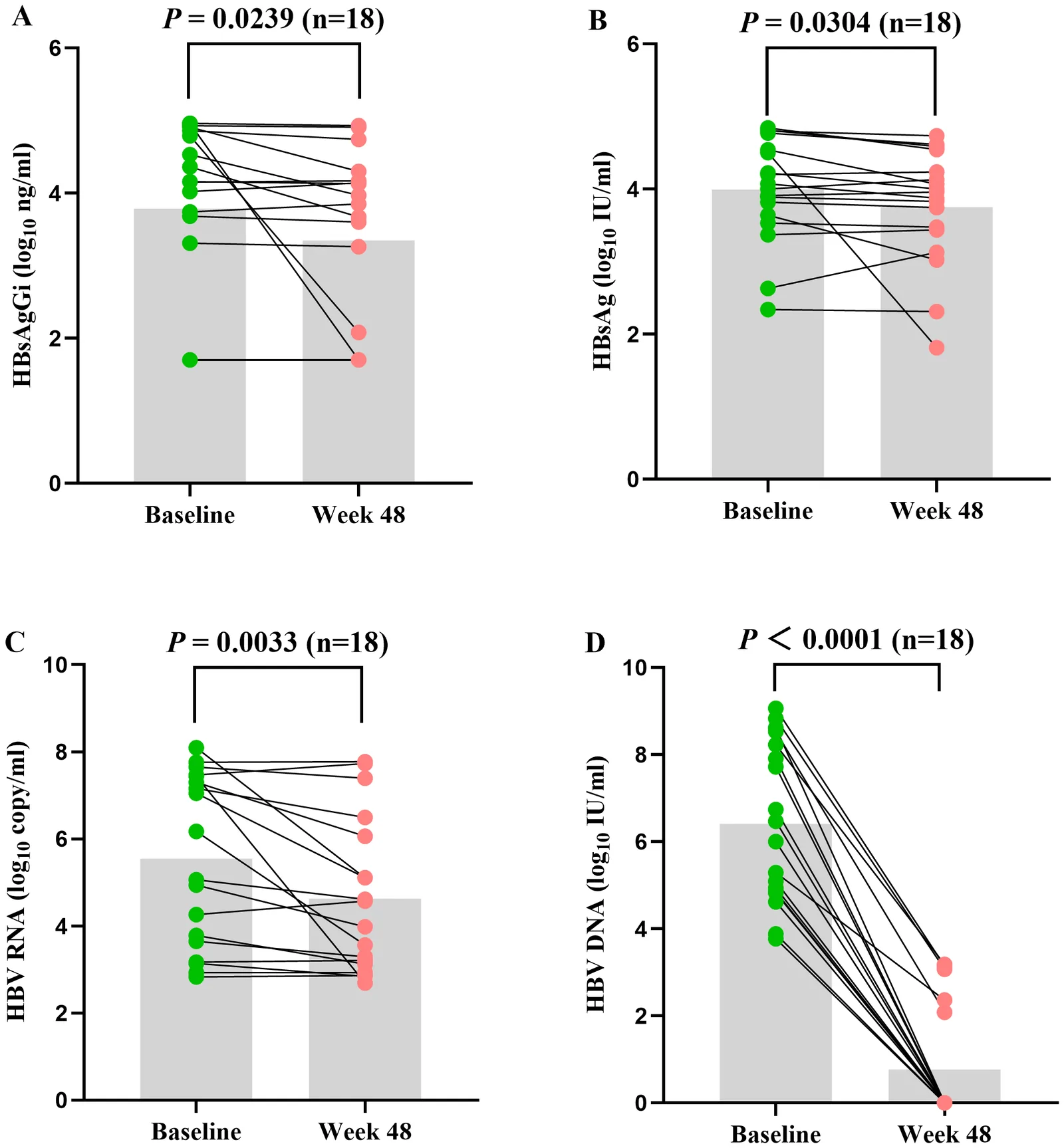
Longitudinal changes of serum viral markers during 48 weeks of treatment. Paired scatter plots showing individual changes in serum levels of HBsAgGi **(A)**, HBsAg **(B)**, HBV RNA **(C)**, and HBV DNA **(D)** from baseline to week 48 in 18 patients. Green dots represent baseline levels, red dots represent week 48 levels, and black lines connect paired samples from the same patient. Gray bars indicate the mean (or median) levels at each time point. P-values indicate the statistical significance of the differences between baseline and week 48.

**Figure 8:**
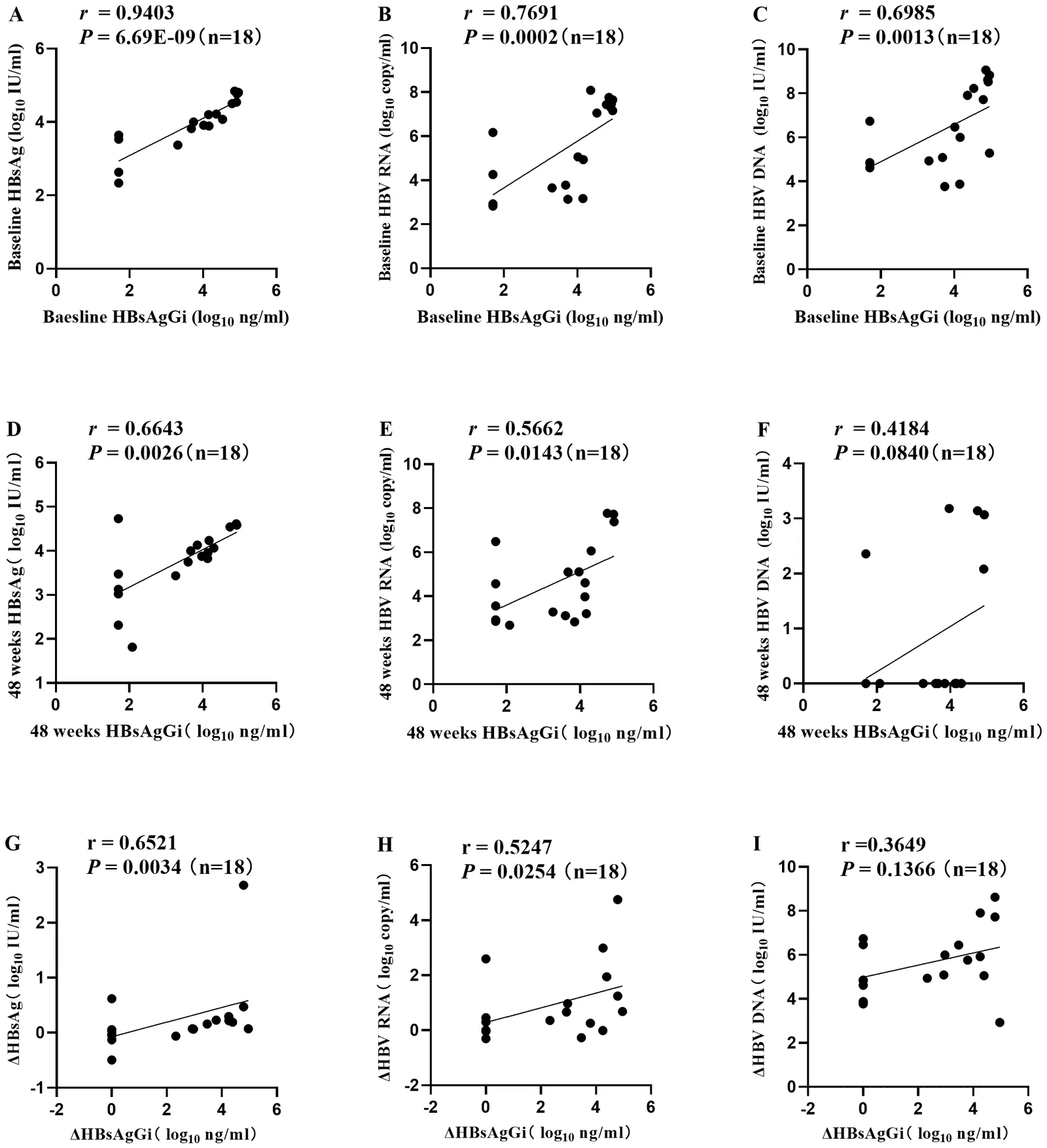
Correlations of serum HBsAgGi with other serum viral markers at baseline, week 48, and their dynamic changes. Scatter plots showing Spearman correlation analyses between serum HBsAgGi and other virological markers. **(A–C)** Correlations at baseline between HBsAgGi and conventional HBsAg **(A)**, HBV RNA **(B)**, and HBV DNA **(C)**. **(D–F)** Correlations at week 48 between HBsAgGi and HBsAg **(D)**, HBV RNA **(E)**, and HBV DNA **(F)**. **(G–I)** Correlations between the changes (Δ) from baseline to week 48 in HBsAgGi and the corresponding changes in HBsAg **(G)**, HBV RNA **(H)**, and HBV DNA **(I)**. All measurements are expressed as log_10_ values. Correlation coefficients (r) and P-values are displayed in each panel (n=18).

Longitudinal evaluation was performed in 18 patients who completed ≥48 weeks of NA therapy, with baseline demographic and clinical characteristics summarized in **Table 2**. Comparative analysis of pre- and post-treatment serum samples revealed a significant decline in HBsAgGi levels from baseline to week 48 (**Table 2**; **Figure 7A**). Concurrent longitudinal monitoring of conventional virological markers revealed parallel significant decreases in HBsAg (**Figure 7B**), HBV RNA (**Figure 7C**), and HBV DNA (**Figure 7D**), with HBV DNA displaying the most pronounced magnitude of decline (**Table 2**). To comprehensively characterize the evolution of interrelationships between HBsAgGi and standard HBV markers under antiviral suppression, we performed correlation analyses focusing on both absolute levels and treatment-induced changes. First, cross-sectional analysis of absolute levels showed that HBsAgGi exhibited robust positive correlations with HBsAg (**Figure 8A**), HBV RNA (**Figure 8B**), and HBV DNA (**Figure 8C**) at baseline. After 48 weeks of NA therapy, these associations showed differential persistence: correlations with HBsAg (**Figure 8D**) and HBV RNA (**Figure 8E**) remained significant, whereas the HBsAgGi-HBV DNA relationship became non-significant (**Figure 8F**). Furthermore, correlation analysis of treatment-induced delta values demonstrated that the reduction in HBsAgGi at week 48 positively correlated with declines in HBsAg (**Figure 8G**) and HBV RNA (**Figure 8H**), but not with HBV DNA reduction (**Figure 8I**).

**Table 2:** Characteristics of 18 patients at baseline and 48 weeks after NAs therapy.

Characteristics	Baseline	48 weeks	*P*-value
Age (years)	40 (37 - 46)	41 (38 - 47)	**-**
Sex (male)	11 (61.1%)	11 (61.1%)	–
ALT (U/L)	42.5 (28 - 72.75)	22.5 (21 - 29)	**0.0002** ^a^
AST (U/L)	26.5 (20 - 39)	22 (17.75 - 24)	**0.0027** ^a^
Albumin (g/L)	45.45 (42.78 - 46.03)	46.2 (43.9 - 48.6)	**0.0317** ^a^
Total bilirubin (µmol/L)	13.85 (12.08 - 15.93)	15.6 (9.08 - 19.9)	>0.9999^a^
Platelet count (×10^9^/L)	185 (137 - 219.5)	200.5 (142.25 - 237)	0.1168^a^
HBsAg (log_10_ IU/ml)	4.03 (3.61 - 4.60)	3.92 (3.36 - 4.31)	**0.0304** ^a^
HBV DNA (log_10_ IU/ml)	6.23 (4.85 - 8.30)	0.00 (0.00 - 2.15)	**<0.0001** ^a^
HBV RNA (log_10_ copies/ml)	5.61 (3.53 - 7.44)	4.27 (3.07 - 6.17)	**0.0033** ^a^
HBsAgGi (log_10_ ng/mL)	4.16 (2.91 - 4.88)	3.76 (1.70 - 4.20)	**0.0239** ^a^
HBeAg (log_10_ S/CO)	1.09 (-0.32 - 3.10)	0.53 (-0.38 - 2.87)	**0.0150** ^a^

Data are expressed as median (interquartile range, IQR), except for sex which is expressed as number (percentage). HBsAgGi, hepatitis B surface antigen glycan isomer; ALT, alanine aminotransferase; AST, aspartate aminotransferase; HBsAg, hepatitis B surface antigen; HBeAg, hepatitis B e antigen; VR, virological response.NA, not applicable.

^a^ Mann Whitney U test. Bold values indicate statistical significance (p < 0.05).

### Predictive value of baseline serum HBsAgGi for virological response to NA therapy

**Figure 9:**
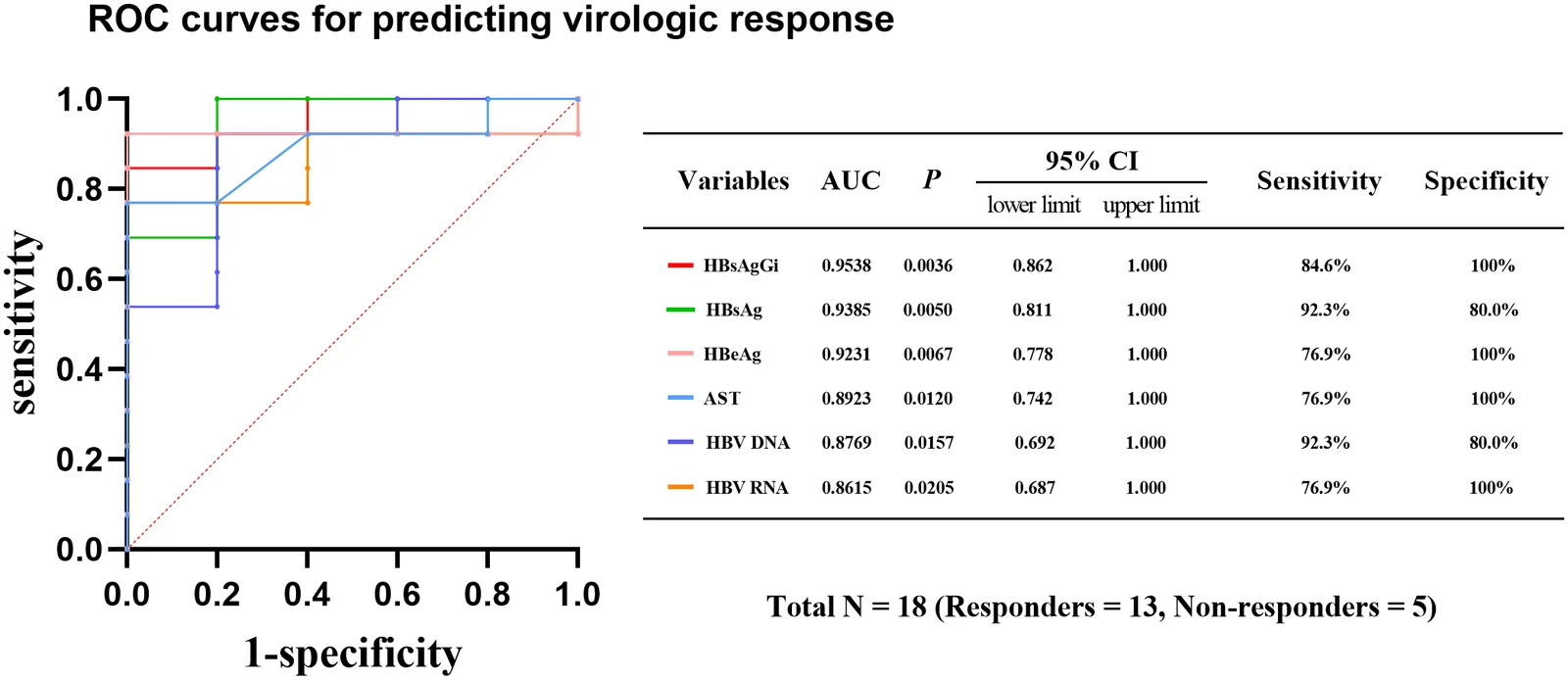
ROC curves of HBsAgGi and other biomarkers at baseline for predicting virological response (VR) after 48 weeks of NA treatment in 18 patients with CHB (VR: N = 13; non-VR: N = 5). ROC curves illustrating the predictive performance of various serum markers (HBsAgGi, HBsAg, HBeAg, AST, HBV DNA, and HBV RNA) for virologic response. The embedded table summarizes the AUC, P-values, 95% CI, Sensitivity and Specificity for each variable. The analysis included a total of 18 patients (13 responders and 5 non-responders).

To identify baseline predictors of VR to NA therapy, we analyzed 18 treated patients, of whom 13 (72.2%) achieved VR at week 48 (**Table 3**). Univariate comparison identified baseline serum HBsAgGi as a significant discriminator of VR status, with responders exhibiting markedly lower levels than non-responders (*P* < 0.01; **Table 3**). ROC curve analysis further quantified the preliminary prognostic utility of HBsAgGi, revealing a high AUC of 0.9538 (95% CI: 0.862 - 1.000) for VR prediction. Notably, HBsAgGi exhibited the numerically highest AUC among all evaluated markers (**Figure 9**). Although the overlapping 95% CI precluded a definitive claim of statistical superiority in this limited cohort, HBsAgGi also demonstrated the most significant difference between VR and Non-VR groups (*P* = 0.0013; **Table 3**). These findings position it as a promising non-invasive predictor for early identification of NA treatment responders, although these findings require validation in larger cohorts.

**Table 3:** Clinical comparisons between VR and non-VR groups.

Characteristics	VR group (n=13)	Non-VR group (n=5)	*P*-value
Age (years)	38 (36 - 47)	40 (37 - 41)	0.9604^a^
Sex (male)	8 (61.5%)	3 (60%)	0.9522^b^
ALT (U/L)	44 (23.5 - 79.5)	32 (27.5 - 42.5)	0.5213^a^
AST (U/L)	27 (22 - 42)	19 (14.5 - 21)	**0.0117** ^a^
Albumin (g/L)	44.3 (42.2 - 46.15)	45.6 (44.45 - 45.8)	0.7024^a^
Total bilirubin (µmol/L)	13.5 (12.25 - 24.25)	14.6 (10.15 - 15.6)	0.7028^a^
Platelet count (×10^9^/L)	175 (133 - 203.25)	210 (155 - 249)	0.2343^a^
HBsAg (log_10_ IU/ml)	3.89 (3.45 - 4.21)	4.8 (4.42 - 4.83)	**0.0028** ^a^
HBV DNA (log_10_ IU/ml)	5.09 (4.72 - 7.23)	8.53 (6.77 - 8.94)	**0.0140** ^a^
HBV RNA (log_10_ copies/ml)	4.26 (3.16 - 6.73)	7.47 (7.11 - 7.70)	**0.0194** ^a^
HBsAgGi (log_10_ ng/mL)	3.68 (1.7 - 4.16)	4.93 (4.7 - 4.96)	**0.0013** ^a^
HBeAg (log_10_ S/CO)	0.26 (-0.25 - 1.97)	3.03 (3.01 - 3.06)	**0.0067** ^a^

Data are expressed as median (interquartile range, IQR), except for sex which is expressed as number (percentage). HBsAgGi, hepatitis B surface antigen glycan isomer; ALT, alanine aminotransferase; AST, aspartate aminotransferase; HBsAg, hepatitis B surface antigen; HBeAg, hepatitis B e antigen; VR, virological response.

^a^ Mann Whitney U test.

^b^ Chi-square test. Bold values indicate statistical significance (p < 0.05).

## Discussion

This study characterized the dynamic correlations linking serum HBsAgGi to conventional and intrahepatic HBV markers throughout the course of NA therapy. Key findings suggest that circulating HBsAgGi can serve as a possible non-invasive surrogate for intrahepatic cccDNA levels and associated transcriptional activity. Importantly, pretreatment HBsAgGi quantification exhibited a promising predictive performance for on-treatment virological response. While its discriminative capacity was comparable to that of conventional HBV markers, HBsAgGi holds potential as a novel, complementary biomarker for evaluating antiviral efficacy.

To our knowledge, this represents the first study comprehensively evaluating serum HBsAgGi against both intrahepatic cccDNA levels and viral transcriptional activity (quantified by intrahepatic HBV-RNA/cccDNA ratio). Current serological markers for intrahepatic cccDNA reservoir and transcriptional activity remain suboptimal. This study focuses on HBsAgGi - a novel biomarker for genotype C HBV infection. The unpublished preclinical data indicate stronger correlation between serum HBsAgGi and intrahepatic cccDNA levels in genotype C HBV-infected mice versus serum HBsAg and HBcrAg (RCMG Inc., Japan). However, clinical evidence in humans was lacking until this study, where we demonstrate that treatment-naïve genotype C CHB patients exhibit significant correlations of serum HBsAgGi levels with intrahepatic cccDNA levels, intrahepatic HBV RNA levels and HBV RNA/cccDNA ratio. Notably, these correlations were stronger than those of conventional markers, suggesting the potential utility of HBsAgGi in monitoring cccDNA reservoir and transcriptional activity. The potential mechanism lies in the fact that HBsAgGi, an O-glycosylated moiety, has been confirmed to exclusively present on the surface of intact, infectious HBV virions containing either HBV DNA or HBV RNA (Wagatsuma et al., 2018). However, conventional HBsAg detection methods use antibodies targeting the common “a” determinant in the S domain shared by all HBsAg isoforms, which cannot differentiate infectious particles from nucleic acid-lacking, non-infectious SVPs (Charre et al., 2019). Therefore, compared to serum HBsAg, serum HBsAgGi may provide a more specific reflection of the level of intact virions and distinguish them from SVPs. Within the liver, in addition to cccDNA serving as the transcriptional and replicative source, there also exists integrated HBV DNA in the host genome (Tu et al., 2021). Integrated HBV DNA enables sustained expression of HBsAg which predominantly assemble into SVPs but cannot generate intact virions whose nucleic acid originates exclusively from cccDNA, thereby uncoupling SVP production from cccDNA activity (Wooddell et al., 2017; Vaillant, 2021). Consequently, HBsAgGi, compared to total HBsAg, may more accurately reflect HBsAg derived from cccDNA. Consistent with this mechanism, our data indicate that serum HBsAgGi demonstrates stronger correlations with intrahepatic cccDNA, HBV RNA, and the RNA/cccDNA ratio compared to total HBsAg.

Our study demonstrates significant correlations between serum HBsAgGi and multiple serum virological markers, including HBV DNA, HBsAg and HBV RNA, validating and extending prior observations (Murata et al., 2022; Kozuka et al., 2024) and the correlation of serum HBsAgGi with HBV RNA reported by Yao et al. (2025) in CHB patients with undetectable HBV DNA at end-of-treatment. The modest correlation between serum HBsAgGi and viral load (HBV DNA and RNA) is likely because HBsAgGi reflects the level of infectious HBV particles, which is not equivalent to serum HBV DNA or RNA that can exist in non-particle-associated forms. More importantly, while serum HBcrAg is widely recognized as a valuable surrogate marker for the intrahepatic cccDNA reservoir (Caviglia et al., 2021; Tu et al., 2022), its correlation with cccDNA in our biopsy subgroup did not reach statistical significance (r = 0.48, *P* = 0.06). This non-significant finding is likely attributable to the limited sample size (n=16) and the resulting reduced statistical power, rather than a true lack of biological association. It is worth noting that while a recent meta-analysis confirmed a significant positive correlation between HBcrAg and cccDNA (r = 0.641, *P* < 0.001), the included studies exhibited substantial heterogeneity (I² = 91.6%) and potential publication bias, indicating that this correlation can be highly variable across different clinical cohorts (Caviglia et al., 2021). Despite these limitations in our small cohort, serum HBsAgGi demonstrated a strong and significant correlation with intrahepatic cccDNA (r = 0.7172, *P* < 0.01) within the same patients. This within-cohort performance highlights the potential of HBsAgGi as a sensitive surrogate marker for the cccDNA reservoir. It is well-established that serum HBV RNA originates from dual sources: replication-dependent full-length pgRNA and replication-independent transcripts derived from integrated HBV DNA, including id-RNAs and 5’-human-HBV-3’RNAs (Zaiets et al., 2023). The stronger concordance between HBsAgGi and intrahepatic cccDNA parameters, relative to HBV RNA, likely reflects HBsAgGi’s preferential derivation from cccDNA-driven transcriptional activity. This might be because a fraction of O-glycosylated M-HBsAg is also present on the surface of SVPs (Wagatsuma et al., 2018). Consequently, this dilutes the correlation between serum HBsAgGi levels and intrahepatic cccDNA transcriptional activity compared to HBcrAg. Clearly, these findings warrant further investigation in the future, such as analyzing the longitudinal correlations between serum HBsAgGi and intrahepatic cccDNA dynamics during and after antiviral therapy to validate its clinical utility for monitoring viral reservoir activity.

Through longitudinal assessment at baseline and week 48 of NA therapy, we revealed three key dynamics. 1) HBsAgGi reduction correlated with declines in HBsAg and HBV RNA, 2) HBsAgGi exhibited slower clearance than HBV DNA, and 3) HBsAgGi maintained post-treatment correlations with HBsAg and HBV RNA but dissociated from HBV DNA, suggesting partial origin from non-Dane particle sources during viral suppression, which is consistent with the previous study (Yao et al., 2025). Additionally, following antiviral treatment, viral replication is suppressed, leading to a significant decline in serum HBV DNA levels. However, the impact on intrahepatic cccDNA levels and its transcriptional activity is often less pronounced during this phase (Tu et al., 2022). Consequently, the correlation between serum HBV DNA and intrahepatic cccDNA becomes weaker under these conditions. This finding is consistent with our observations in the overall cohort and is likely attributable to the aforementioned scarcity of infectious HBV particles in these patients. Lastly, baseline HBsAgGi predicted virological response with favorable accuracy, in line with findings from prior research (Yao et al., 2025), showing improved predictive performance compared to conventional markers and demonstrating potential clinical value as a treatment response indicator. These findings collectively suggest that HBsAgGi may serve as a comprehensive biomarker for monitoring genotype C-specific viral activity and therapeutic efficacy.

Despite the limitations of sample size, this study provides the first clinical evidence supporting the dual utility of HBsAgGi as a surrogate for intrahepatic cccDNA transcriptional activity and a monitor for residual viral burden. In patients undergoing long-term NA therapy, conventional markers often lose their discriminatory power: serum HBV DNA is frequently suppressed below detection limits, masking residual cccDNA transcription (Lampertico et al., 2017). Total HBsAg remains elevated due to integrated HBV DNA rather than active replication (Levrero and Zucman-Rossi, 2016; Wooddell et al., 2017), and HBeAg is uninformative in seroconverted patients (Seto et al., 2018). In this specific clinical scenario, HBsAgGi may serve as a valuable complementary tool. By specifically targeting O-glycosylated M-HBsAg derived from transcriptionally active cccDNA (Dane particles), it avoids the interference of “mirror mutations” (such as those in the major hydrophilic region induced by long-term NA therapy) that compromise conventional immunoassays (Ding et al., 2014), offering a potentially more precise assessment of the true viral reservoir.

Several limitations of this study should be acknowledged. First, the modest sample sizes of our specific subcohorts, particularly the liver biopsy (n=16) and treatment (n=18) cohorts, restrict the statistical power and generalizability of our findings. Although we observed consistently notable correlations and consistent longitudinal predictive trends despite the small cohorts, these results must be interpreted with caution. Second, the current study focused exclusively on HBV genotype C. This was because O-glycosylated M-HBsAg is unique to genotype C infectious virions, and specific detection antibodies for genotype B, a major prevalent strain in China, are currently under development.

Despite these limitations, the current study serves as a proof-of-concept that provides initial evidence supporting the potential clinical value of HBsAgGi. Future large-scale, multi-center, prospective studies with long-term follow-up are warranted. These investigations should aim to expand the cohort size to validate the predictive value of HBsAgGi for functional cure, and incorporate diverse HBV genotypes (such as genotype B) once the corresponding assays become available, thereby fully establishing its broad applicability across different disease stages and viral genotypes.

## Data Availability

The datasets presented in this study can be found in online repositories. The names of the repository/repositories and accession number(s) can be found in the article/**Supplementary Material**.
