# Supplementary material for: HBsAg glycan isomer: a potential circulating biomarker for monitoring cccDNA and predicting virologic response in chronic hepatitis B
Source: Front Cell Infect Microbiol. 2026 Aug 20;16:1818958. doi: 10.3389/fcimb.2026.1818958 (PMC13537954; doi:10.3389/fcimb.2026.1818958)
Supplement: Supplementary file 1 [file DataSheet1.docx]

**Supplementary Figure 1. Flowchart of the study population and subcohorts.** The overall cohort consisted of 73 untreated CHB patients with HBV genotype C. Patients were stratified into specific subgroups for analysis: the HBcrAg subcohort (n = 28), which included the Liver Biopsy subcohort (n = 16) as a subset; the Treatment subcohort (n = 18); and patients not included in specific subcohorts (n = 27).

**Supplementary Figure 2. Principle of HBsAgGi antibody capture for infectious HBV enrichment.** The schematic distinguishes infectious DNA virions (Dane particles containing nucleocapsid and O-glycosylated M-HBsAg) from the abundant subviral particles (SVPs) in patient serum (left). Through specific antibody capture targeting HBsAgGi (middle), infectious Dane particles are successfully enriched and isolated from SVPs (right).

**Supplementary Figure 3. Correlation between serum HBsAgGi levels and serum virological markers in the HBeAg-positive group (n=20) of the untreated CHB overall cohort.** Scatter plots showing Spearman correlation analyses between serum HBsAgGi and conventional HBsAg (A), HBV DNA (B), HBV RNA (C), and HBeAg (D). All measurements are expressed as log₁₀ values. Correlation coefficients (r) and P-values are displayed in each panel.

**Supplementary Figure 4. Correlation between serum HBsAgGi levels and virological markers in the HBeAg-negative group (n=53) of the untreated CHB cohort.** Scatter plots showing Spearman correlation analyses between serum HBsAgGi and conventional HBsAg (A), HBV DNA (B), HBV RNA (C), and HBeAg (D). All measurements are expressed as log₁₀ values. Correlation coefficients (r) and P-values are displayed in each panel.

**Supplementary Figure 5. Correlations between serum HBV DNA and intrahepatic viral markers.** Scatter plots showing Spearman correlation analyses between serum HBV DNA levels and intrahepatic cccDNA (A), intrahepatic HBV RNA (B), and the ratio of intrahepatic RNA/cccDNA (C). All measurements are expressed as log₁₀ values. Correlation coefficients (r) and P-values are displayed in each panel.

| **Supplementary Table 1. Characteristics of 16 patients with liver biopsy.** | |  |
| --- | --- | --- |
| **characteristic** | **All patients (n=16)** | |
| Age (years) | 52 (37 - 58) | |
| Sex (male) | 9 (56.3%) | |
| ALT (U/L) | 32 (22.5 - 50.25) | |
| AST (U/L) | 24.5 (18.75 - 36.75) | |
| Albumin (g/L) | 45.5 (42.55 - 45.9) | |
| Total bilirubin (µmol/L) | 16.7 (12.83 - 19.5) | |
| Platelet count (×10^9^/L) | 203 (150 - 244.5) | |
| HBsAg (log_10_ IU/ml) | 3.26 (1.43 - 3.77) | |
| HBV DNA (log_10_ IU/ml) | 2.7 (0.42 - 3.86) | |
| HBV RNA (log_10_ copies/ml) | 3.29 (2.76 - 4.24) | |
| HBcrAg (log_10_ IU/ml) | 3.15 (2.24 - 4.58) | |
| HBsAgGi (log_10_ ng/ml) | 2.61 (2.25 - 3.42) | |
| HBeAg (log_10_ S/CO) | -0.46 (-0.52 - -0.23) | |
| HBeAg positivity | 2 (12.5%) | |
| Intrahepatic cccDNA (log_10_ copies/10^6^ cells) | 4.93 (4.74 - 5.47) | |
| Intrahepatic HBV RNA (log_10_ copies/10^6^ cells)  Liver-RNA（log_10_ copies/10^6^cell）  Liver-RNA（log_10_ copies/10^6^cell） | 4.22 (3.99-5.30) ** | |
| Intrahepatic HBV RNA/cccDNA ratio | 0.29 (0.12-0.40) | |
| Data are expressed as median (interquartile range, IQR), except for sex and HBeAg positivity, which are expressed as numbers (percentages). Abbreviations: HBsAgGi, hepatitis B surface antigen glycan isomer; ALT, alanine aminotransferase; AST, aspartate aminotransferase; HBsAg, hepatitis B surface antigen; HBeAg, hepatitis B e antigen; cccDNA, covalently closed circular DNA.  **The level was below the lower detection limit in three patients. | | |

| **Supplementary Table 2. Characteristics of 28 patients with HBcrAg levels.** | |
| --- | --- |
| **Characteristics** | **All patients (n=28)** |
| Age (years) | 41 (35 - 50) |
| Sex (male) | 18 (64.3%) |
| ALT (U/L) | 23 (19 - 54.25) |
| AST (U/L) | 19.5 (17 - 30.5) |
| Albumin (g/L) | 45.8 (44.5 - 47.98) |
| Total bilirubin (µmol/L) | 13.4 (9.65 - 19.85) |
| Platelet count (×10^9^/L) | 202 (164 - 269) |
| HBsAg (log_10_ IU/ml) | 3.21 (2.19 - 3.62) |
| HBV DNA (log_10_ IU/ml) | 2.91 (2.4 - 3.63) |
| HBV RNA (log_10_ copies/ml) | 1.7 (0.0 - 2.8) |
| HBcrAg (log_10_ U/mL) | 3.29 (2.17 - 4.23) |
| HBeAg (log_10_ S/CO) | -0.22 (-0.24 - -0.19) |
| HBeAg positivity | 3 (10.7%) |
| HBsAgGi (log_10_ ng/mL) | 2.83 (2.22 - 3.67) |
| Data are expressed as median (interquartile range, IQR), except for sex and HBeAg positivity, which are expressed as numbers (percentages). Abbreviations: HBsAgGi, hepatitis B surface antigen glycan isomer; ALT, alanine aminotransferase; AST, aspartate aminotransferase; HBsAg, hepatitis B surface antigen; HBeAg, hepatitis B e antigen; HBcrAg, hepatitis B core-related antigen. | |
